# Reviewers

**DOI:** 10.1111/jdi.13718

**Published:** 2021-12-17

The publication of invaluable papers in the Journal of Diabetes Investigation depends on the prompt, careful review of submitted manuscripts. We would like to thank the Editorial Board members, the International Editorial Board members, the Assistant Editorial Board members and the following experts for reviewing manuscripts from September 1, 2020 to August 31, 2021.

Masanori Abe

Norio Abiru

Clifton Addison

Ernest Adeghate

Vasudha Ahuja

Jun Aida

Toru Aizawa

Sadanori Akita

Gulali Aktas

Sirajudeen S. Alavudeen

Nasser M. Al‐Daghri

Rachael Allen

Keizo Anzai

Shigeru Aoki

Keiko Arai

Atsushi Araki

Shin‐ichi Araki

Noriko Asahara

Shunichiro Asahara

Yoshimasa Aso

Dagfinn Aune

Masayuki Baba

Tetsuya Babazono

Megu‐Yamaguchi Baden

Yuqian Bao

Neal Barshes

Arnaud Del Bello

Maria Bonsignore

Ryotaro Bouchi

David S. Boyer

Guoqi Cai

Xiaoling Cai

Soner Cander

Luke Carroll

Ali J. Chakera

Subrata Chakrabarti

Juliana Chan

Te‐Fu Chan

Jer‐Ming Chang

Nai‐Jen Chang

Chia‐Ter Chao

Cheng‐Hsu Chen

Gin‐Shin Chen

Harn‐Shen Chen

Shi Chen

Yu‐Ju Chen

Ken Chiu

Sung Hee Choi

Chih‐Hsun Chu

NF Chu

Daisuke Chujo

Dana Ciobanu

Heiner Claessen

Makoto Daimon

Masao Daimon

Daisuke Taura

Undurti Das

Buyzere Marc De

Takahisa Deguchi

Kentaro Doi

Nathan Efron

Masanori Emoto

Fabio Fabbian

Kang‐Chih Fan

Yaofu Fan

Jose L. Flores‐Guerrero

Toshihito Fujii

Rumi Fujikawa

Kei Fujimoto

Shimpei Fujimoto

Yoshio Fujioka

Shiho Fujisaka

Tomomi Fujisawa

Yoshihito Fujita

Yukihiro Fujita

Yoshio Fujitani

Yukio Fujiwara

Kei Fukami

Astunori Fukuhara

Michiaki Fukui

Tomoyasu Fukui

Nobuaki Funahashi

Masato Furuhashi

Kenji Furukawa

Shinya Furukawa

Hiroto Furuta

Mauro Gacci

You‐Shui Gao

Carmine Gazzarusp

Abed Ghavami

Stacey Gorniak

Atsushi Goto

Ali Erdal Gunes

Yashdeep Gupta

Kyoung Hwa Ha

Seiya Hagiwara

Yoji Hamada

Kazuyuki Hamaguchi

Masahide Hamaguchi

Tomoya Hamaguchi

Akihiro Hamasaki

Toshiaki Hanafusa

Akinori Hara

Mariko Harada‐Shiba

Ai Harashima

Naotake Hashimoto

Mohamed Hassanein

Yuji Hataya

Tsuyoshi Hattori

Akinori Hayashi

Yoshitaka Hayashi

Yasuaki Hayashino

Finn Henriksen

Mudiyanselage Meththananda Herath

Tatsuhito Himeno

Yoichiro Hirakawa

Takumi Hirata

Takahisa Hirose

Yushi Hirota

Low‐Tone Ho

Tomohiro Hori

Ichiro Horie

Takeshi Horii

Yukio Horikawa

Masayuki Hosoi

Ryota Hosomi

Chang‐Hsun Hsieh

Cheng Hu

Ji Hu

Wei Syun Hu

Chia‐Luen Huang

Chun‐Feng Huang

CN Huang

Mikael S Huhtala

Tai‐Ho Hung

You‐Cheol Hwang

Chii‐Min Hwu

Jon Hyett

Yasuo Ido

Katsumi Iizuka

Hiroshi Ikegami

Satoru Ikenoue

Junta Imai

Minako Imamura

Takeshi Inagaki

Junichiro Irie

Fukashi Ishibashi

Yasushi Ishigaki

Hisamitsu Ishihara

Yoshiya Ito

Katsuomi Iwakura

Naoko Iwasaki

Masanori Iwase

Minoru Iwata

Anwar Ali Jammah

Hak Chul Jang

Edward Janus

Peter Jarcuska

Troels Staehelin Jensen

Ja Young Jeon

Jae‐Han Jeon

Guozhi Jiang

Sang‐Man Jin

Hideaki Jinnouchi

Inha Jung

Dianna Juvinao‐quintero

Elnaggar Nabil K.

Tetsuya Kakuma

Naotetsu Kanamoto

Keizo Kanasaki

Akio Kanazawa

Ippei Kanazawa

Shizuka Kaneko

Hideaki Kaneto

Atsunori Kashiwagi

Naoto Katakami

Hisaya Kato

Koichi Kato

Yoshiro Kato

Yuji Kawaguchi

Tomoko Kawai

Dan Kawamori

Daiji Kawanami

Eiji Kawasaki

Haseeb A. Khan

Madhu Khullar

Ishant Khurana

Yoshiaki Kido

Takeshi Kikuchi

Toru Kikuchi

Chong Hwa Kim

Jin Taek Kim

Kyung‐Soo Kim

Nan Hee Kim

Sang Soo Kim

Sang Yong Kim

Miyako Kishimoto

Munehiro Kitada

Tadahiro Kitamura

Yuzo Kodama

Tomomi Kogiso

Takuma Kondo

Tatsuya Kondo

Masaya Koshizaka

Ahmed F. Kotb

Daisuke Koya

Hidenori Koyama

Igor Kozak

Junji Kozawa

Naoto Kubota

Shinji Kume

Ching‐Hua Kuo

Chun‐Heng Kuo

Akio Kuroda

Yoshiki Kusunoki

Hitoshi Kuwata

Tsuo‐Hung Lan

Meng Lee

Da Young Lee

Dae Ho Lee

Eun Young Lee

I‐Te Lee

Jen‐Kuang Lee

Jie‐Eun Lee

Ji‐Hyun Lee

Mei‐Yueh Lee

Seung Hwan Lee

Ting‐Wei Lee

Won‐Young Lee

Yau‐Jiunn Lee

Yongho Lee

Li Yu‐Huei

hong‐yuan li

Xia Li

Yuanying LI

Yuxiu Li

Zongjin Li

Kuan‐Fu Liao

Tang‐Yi Liao

Xinxue Liao

Cadmon Lim

Soo Lim

Chia‐Huei Lin

Chia‐Hung Lin

Hugo You‐Hsien Lin

Kun‐Der Lin

Liang‐Yu Lin

Shi‐Dou Lin

Shih‐Yi Lin

Shin‐Jong lin

Shin‐Yu Lin

Jian‐Ping Liu

Jing‐Ying Lu

Peirong Lu

Qingxian Luan

David Lui

Jianhua Ma

Katarzyna Madziarska

Shiro Maeda

Loey Mak

Noboru Makabe

Makoto Makishima

Rayaz Malik

Krzysztof Marycz

Takayuki Masaki

Yamanouchi Masayuki

Tandi Edith Masha

Hiroaki Masuzaki

Ikuro Matsuba

Masafumi Matsuda

Munehide Matsuhisa

Munetaka Matsuhisa

Takeshi Matsumura

Naoki Matsuoka

Taka‐aki Matsuoka

Takashi Matsuoka

Takashi Miki

Takayuki Miki

María Miranda

Tomoya Mita

Junnosuke Miura

Takeshi MIyatsuka

Hideaki Miyoshi

Hiroki Mizukami

Mie Mochizuki

Jun Sung Moon

Katsuhito Mori

Yasumichi Mori

Kastutaro Morino

Tomoaki Morioka

Carmine Morisco

Yoshiaki Morishita

Yuki Morizane

Wali Muhammad

Chieko Murakami

Takaaki Murakami

Tomoaki Murakami

Koji Murao

Takashi Murata

Serafin Murillo

Yoshio Nagai

Mototsugu Nagao

Shoichiro Nagasaka

Terumasa Nagase

Masao Nagata

Tomoko Nakagami

Kei Nakajima

Akinobu Nakamura

Nobuhisa Nakamura

Shuhei Nakanishi

Takao Nammo

Koji Naruishi

Getandale Zeleke Negera

Dinesh Neupane

Yuichiro Nishida

Norihisa Nishimura

Rimei Nishimura

Wataru Nishimura

Mitsuhiko Noda

Yoshihiko Noma

Takashi Nomiyama

Takuo Nomura

Shinsuke Noso

Makiko Ogata

Sumito Ogawa

Masahito Ogura

Tae Jung Oh

Koji Ohashi

Toshiaki Ohkuma

Haruya Ohno

Mitsuru Ohsugi

Masayuki Ohta

Yasuharu Ohta

Yoichi Oikawa

Rie Oka

Shinichi Oka

Yosuke Okada

Yoshiaki Okubo

Yukiko Onishi

Yoshinori Osaki

Vahidreza Ostadmohammadi

Nobuaki Ozaki

Nikolaos Papanas

Hyeong Kyu Park

Tianqing Peng

Elisabetta Poluzzi

Stergios Polyzos

Louis Potier

Sharad Purohit

Lin Qi

Ajenthen Ranjan

Marc Rendell

Eun‐Jung Rhee

Ewa Romejko‐Wolniewicz

Shuang Rong

Leanna Ross

Alicja R. Rudnicka

Yoshifumi Saisho

Kazuhiko Sakaguchi

Mashito Sakai

Hiroshi Sakaue

Masaru Sakurai

Kazunori Sango

Giorgio Maria Saracco

Hideyuki Sasaki

Toshiyasu Sasaoka

Yuzo Sato

Hiroaki Satoh

Jo Satoh

Dinesh Selvarajah

Takafumi Senokuchi

Viral N. Shah

Jonathan E. Shaw

Yunfeng Shen

Meei‐Ling Sheu

Kenichi Shikata

Michio Shimabukuro

Akira Shimada

Ikki Shimizu

Masashi Shimoda

Seiya Shimoda

Dai Shimono

Kei Shinoda

Jun Shirakawa

Takuhito Shoji

Ki‐Ho Song

Kee‐Ho Song

Yiqing Song

Ian Spence

Norbert Stefan

Elena Succurro

Kazuhiro Sugimoto

Ken Sugimoto

Masahiko Sugimoto

Takashi Sugiyama

Sunghwan Suh

Zilin Sun

Junichi Suzuki

Ken Suzuki

Ryo Suzuki

Yasuharu Tabara

Yuji Tajiri

Mitsuyoshi Takahara

Kazuma Takahashi

Masaaki Takamura

Toshinari Takamura

Fumihiko Takeuchi

Yasuo Takeuchi

Yoshikazu Tamori

Yoshifumi Tamura

Elise Chia‐Hui Tan

Daisuke Tanaka

Nagaaki Tanaka

Tomohiro Tanaka

Hatice Tankisi

Shimosawa Tatsuo

Yasuo Terauchi

Xiao Yu Tian

KJ Tien

Kazuyuki Tobe

Atsuhito Tone

Nanwei Tong

Serena Tonstad

Keiichi Torimoto

Satoru Tsujii

Tetsuro Tsujimoto

Shin Tsunekawa

Yu‐Kang Tu

Jaakko Tuomilehto

Yasuko Uchigata

Hiroaki Ueno

Satoshi Ugi

Ibtehaj Ul‐Haque

Tatsuhiko Urakami

Emi Ushigome

Isao Usui

Ryota Usui

Takashi Uzu

Francesc Villarroya

Jun Wada

Jackie Walejko

Chaofan Wang

Chih Yuan Wang

Jann‐Yuan Wang

Jun‐Sing Wang

Niansong Wang

PH Wang

Shu‐Huei Wang

Xiao Qun Wang

Mitsuhiro Watanabe

Paweł Wiech

Jong Chul Won

Chung‐Ze Wu

Wang‐Hong Xu

Zhangrong Xu

Kunimasa Yagi

Naoya Yahagi

Satoru Yamada

Kazuya Yamagata

Sho‐ichi Yamagishi

Takeshi Yamamoto

Yasuhiko Yamamoto

Shunsuke Yamane

Yuji Yamazaki

T‐Y Yang

Xilin Yang

Xuefeng Yang

Yeoree Yang

Hisafumi Yasuda

Ichoro Yasuhi

Hiroshi Yatsuya

Hiroshi Yokomichi

Sho Yoneda

Takashi Yoneda

Hideto Yonekura

Shin Yonemitsu

Tohru Yorifuji

Bedowra Zabeen

Sen Zhang

Yang Zhang

Yongliang Zhang

Yu Zhang

Jian Zhou

Naiming Zhou

